# Tuning the Composition of AuPt Bimetallic Nanoparticles for Antibacterial Application**

**DOI:** 10.1002/anie.201401035

**Published:** 2014-05-14

**Authors:** Yuyun Zhao, Chunjie Ye, Wenwen Liu, Rong Chen, Xingyu Jiang

**Affiliations:** Beijing Engineering Research Center for BioNanotechnology, CAS Key Lab for Biological Effects of Nanomaterials and Nanosafety, National Center for NanoScience and Technology11 Beiyitiao, ZhongGuanCun, Beijing 100190 (China); Wuhan Institute of TechnologyXiongchu Avenue, Wuhan, 430073 (China)

**Keywords:** bacteria, bimetallic nanoparticles, gold, platinum

## Abstract

We show that bimetallic nanoparticles (NPs) of AuPt without any surface modification are potent antibiotic reagents, while pure Au NPs or pure Pt NPs display no antibiotic activities. The most potent antibacterial AuPt NPs happen to be the most effective catalysts for chemical transformations. The mechanism of antibiotic action includes the dissipation of membrane potential and the elevation of adenosine triphosphate (ATP) levels. These bimetallic NPs are unique in that they do not produce reactive oxygen species as most antibiotics do. Being non-toxic to human cells, these bimetallic noble NPs might open an entry to a new class of antibiotics.

Some metals such as Ag and Hg are well known to have intrinsic antibacterial properties.^1^ But these metals also pose serious threat to humans as they are toxic to human cells. Several reports have resorted to using nanoparticles (NPs) made of less toxic metals, such as gold, as safe antibiotics. Even though considered biologically inert in its bulk state, gold in the form of NPs can be activated as antibiotics by modifying with functional organic molecules on their surfaces.^2^ Because surface modification brings complications in characterization and preparation, we have been seeking NPs of nontoxic metals that do not require surface modifications to use as antibiotics. Our initial screening shows that single-component non-toxic metallic NPs do not have antibiotic capability;2a we thus turn to bimetallic NPs. While bimetallic NPs have found wide uses in chemical catalysis,^3^ nothing is known about their biological properties. AuPt NPs can catalyze many types of reactions, for example, the oxidation of methanol,^4^ ethanol,4b glycerol,^5^ other alcohols,^6^ formic acid,4b and glucose.3d The catalytic activity is highly dependent on the composition and the atomic arrangement.^7^ AuPt NPs show the best activity with a Pt content between 15–35 % for methanol oxidation,4b, 7b between 20–40 % for oxygen reduction,^8^ and between 28–40 % Pt for glucose oxidation.^9^ Most biological reactions are completed with the aid of enzymes. We hypothesized that the catalytic capability of bimetallic NPs at mild conditions could result in novel biological activities via effects on enzymatic activities (or the catalytic activities in cells). Here, we examined the viability of bacteria and mammalian cells under the influence of AuPt NPs, the morphological change of bacterial cells, and the respiration process to show that AuPt NPs can be potent antibiotics.

We synthesized AuPt NPs by the co-reduction of HAuCl_4_ and K_2_PtCl_4_ with sodium borohydride using Tween 80 as a stabilizer in water in an ice-water bath. We quantified the composition of bimetallic NPs using inductively coupled plasma-optical emission spectroscopy (ICP-OES) and observed their morphology using transmission electron microscopy (TEM). Monometallic NPs were synthesized using only the corresponding salt. These NPs were 2–3 nm in diameter (Figure S1 in the Supporting Information). The characteristic UV/Vis absorption of Au NPs disappeared with the increase of the Pt content (Figure S2). AuPt NPs were negatively charged in water (Table S1), which agrees with reported results.^10^ When the content of Pt was larger than 50 %, the negative charge of NPs increased with the increase of Pt.

We evaluated the antibacterial activities of AuPt NPs with the minimal inhibitory concentration (MIC) according to the reported method.2b AuPt NPs show significant antibiotic activities when the Pt content is between 10 % and 65 % (Table 1). The tested bacteria include five most important clinically Gram-negative bacteria, *Escherichia coli* (*E. coli*), multidrug-resistant *E. coli* (MDR *E. coli*), *Pseudomonas aeruginosa* (*P. a*), *Klebsiella pneumoniae* (*K. p*), and *Salmonella choleraesius* (*S. c*). Gram-negative bacteria can induce the infection of almost all organs in body.^11^ *E. coli* and *K. p* can induce the infection of urinary, biliary, gastrointestinal tracts, lung, and blood. *P. a* and *K. p* can induce lung infection. *E. coli* and *S. c* can cause severe food contamination. MDR *E. coli* was isolated from a local hospital and carried extended-spectrum β-lactamase (ESBL) resistance genes. In contrast to bimetallic NPs, monometallic Au and Pt NPs showed no antibacterial activity against all tested bacteria. AuPt NPs with the Pt content larger than 65 % showed weak activities and those with the Pt content of 94 % had no activity. AuPt NPs with the Pt content between 10–65 % showed extensive antibacterial activities against both the laboratory standard strains and the clinical MDR strain. AuPt NPs with 20 % Pt showed the best activity. The MIC was 5 μg mL^−1^ against *E. coli*, MDR *E. coli*, *P. a*, and *K. p*, and 9 μg mL^−1^ against *S. c*. The MIC values thus indicate that the bimetallic AuPt NPs are potent antibiotic reagents.

**Table 1:** Antibacterial activities of AuPt NPs and monometallic NPs (minimal inhibitory concentration, MIC, μg mL^−1^).

Au_100−*x*_Pt_*x*_^[a]^	MIC [μg mL^−1^]				
	*E. coli*^[b]^	MDR *E. coli*	*P. a*	*K. p*	*S. c*
Au	>128	>128	>128	>128	>128
Au_95_Pt_5_	>128	16	>128	>128	>128
Au_94_Pt_6_	>128	5	17	9	34
Au_90_Pt_10_	5	5	9	9	18
Au_80_Pt_20_	5	5	5	5	9
Au_66_Pt_34_	6	6	6	12	12
Au_51_Pt_49_	16	16	16	16	32
Au_35_Pt_65_	23	23	46	46	46
Au_20_Pt_80_	41	>128	82	82	82
Au_6_Pt_94_	>128	>128	>128	>128	>128
Pt	>128	>128	>128	>128	>128
gentamicin	1	>64	2	1	4
levofloxacin	0.12	32	2	0.12	0.12

[a] *x* is the atomic percentage of Pt in the NP. [b] *E. coli*=*Escherichia coli*, *P. a*=*Pseudomonas aeruginosa*, *K. p*=*Klebsiella pneumoniae*, *S. c*=*Salmonella choleraesius*.

We next determined the minimal bactericidal concentrations (MBCs) of *E. coli* to determine if the bimetallic NPs can be bactericidal. MICs indicate the ability of inhibiting the growth of bacteria but not necessarily killing them, while MBCs indicate the ability of antibiotics in killing bacteria.^12^ A bactericidal agent is defined as a material with a ratio of MBC to MIC≤4.12b Antibiotics with a ratio of MBC to MIC>4 are defined as bacteriostatic agents. The bactericidal agent kills bacteria rapidly and reduces the development of bacterial resistance, hence as a better choice for clinicians in most cases.12a The MBCs of AuPt NPs with the Pt content between 10–65 % are the same as their MICs, which means that AuPt NPs belong to bactericidal agents against *E. coli*.

We investigated antibacterial activities of NPs in the presence of biomacromolecules. Biomacromolecules like proteins can adsorb to the surface of NPs and probably change their biological effects.^13^ When we added 10 % fetal bovine serum in the broth, two representative NPs, Au_80_Pt_20_ and Au_66_Pt_34_, showed effective activities against bacteria (Table S2). The presence of biomacromolecules thus does not significantly affect the antibacterial activity of NPs.

We note that the most effective antibiotic AuPt NPs are also the best catalysts reported in the literature (Table S3).4b, 7b, 8a Reports in the catalysis of bimetallic NPs state that the synergistic catalysis originates from the change of electron states for both Au and Pt in AuPt NPs.^7^ The transfer of charge from Pt to Au leads to the increase of d-orbital vacancy in AuPt NPs and the change of the electrocatalytic properties of Au and Pt. When Pt is doped into Au NPs, it increases the adsorption or production of oxygenated species on Au, which is important for catalytic reactions.^7^ Semiconductor–metal composite Ag_2_S/Ag NPs killed bacteria presumably by a local circuit loop and reactive oxygen species (ROS) produced under UV irradiation.^14^ In the following section, we investigate the antibacterial action of AuPt NPs by the observation of bacterial morphology, the detection of membrane permeability, the respiration process (the intracellular redox reaction), and ROS.

We took *E. coli* as an example to investigate the mechanism of action of AuPt NPs. We visualize the morphological change of *E. coli* treated with AuPt NPs using scanning electron microscopy (SEM) and transmission electron microscopy (TEM) (Figure 1). Antibacterial Au_90_Pt_10_, Au_80_Pt_20_, and Au_51_Pt_49_ induced cell lysis (Figure 1 A). TEM images confirmed their structural changes. Au_80_Pt_20_ induced blurring of the cytoplasm membrane boundary, loss of interior structures, and the formation of a large-scale light area (Figure 1 B), which suggests that the lysis of bacterial cells took place.^15^ Hence, AuPt NPs can induce disruption to cell membrane and the lysis of bacterial cells.

**Figure 1:**
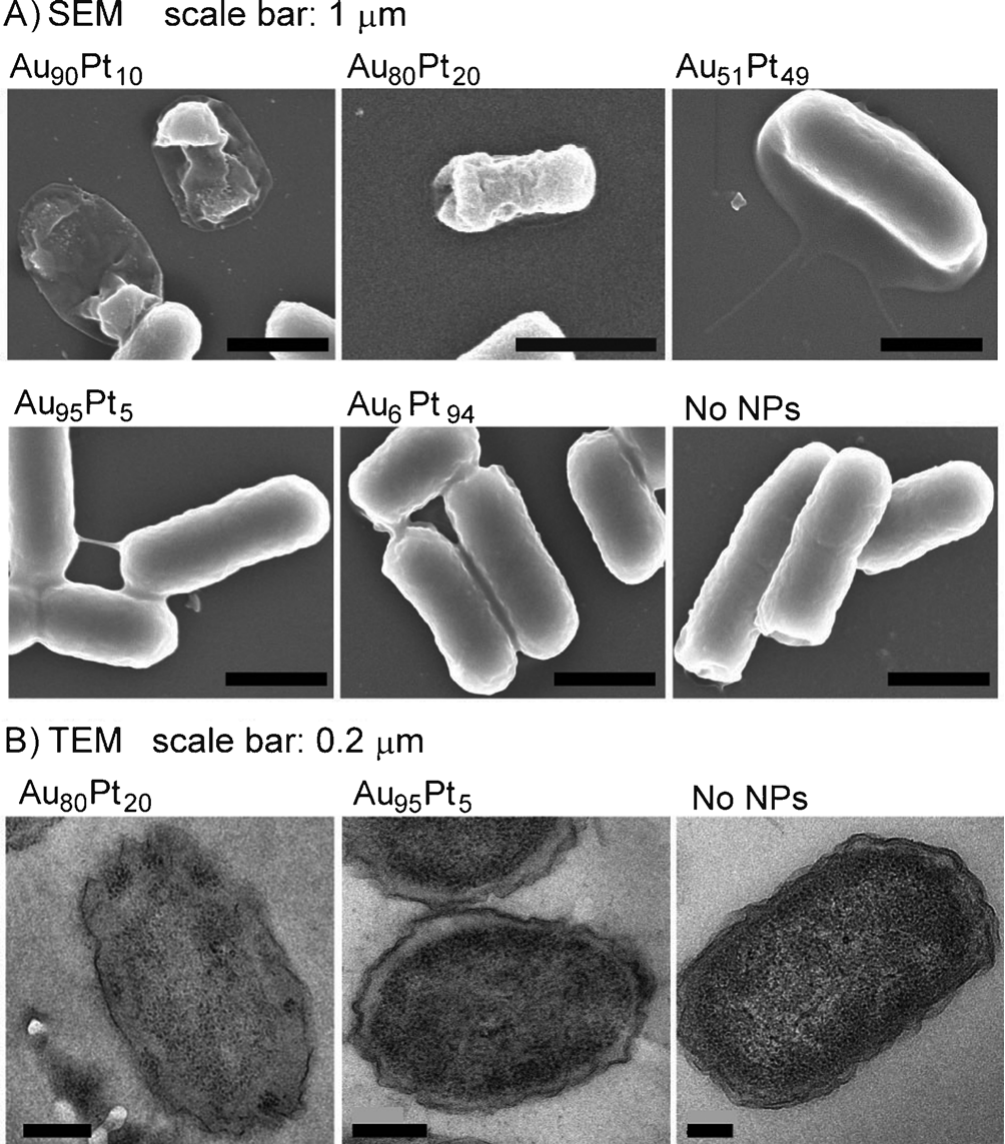
Morphological changes of *E. coli* treated with AuPt NPs (40 μg mL^−1^, 2 h) visualized with A) SEM and B) TEM. In (A), antibacterial Au_90_Pt_10_, Au_80_Pt_20_, and Au_51_Pt_49_ induced the lysis of bacterial cells. In (B), Au_80_Pt_20_ induced blurring of the cytoplasm membrane, loss of the interior structure, and formation of a large-scale light area (the status of lysis).

We used fluorescent dyes to assess the integrity of the cell membrane in the presence of NPs. The hydrophobic fluorophore 1-*N*-phenylnaphthylamine (NPN) can bind to the outer membrane and yields increased fluorescence when bacterial outer membrane is disrupted. Hence, the fluorescent dye can indicate the action of NPs on the outer membrane. We treated *E. coli* with 40 μg mL^−1^ AuPt NPs for 4 h, collected bacterial cells, and incubated them with NPN for 30 min. All of AuPt NPs can increase the fluorescence to some extent (Figure 2 A). We conclude that the structural change of outer membrane could not be a cause for the antibacterial action of AuPt NPs with 10–65 % of Pt. We used the dye DiSC_3_(5) to probe the inner membrane potential because the fluorescence of the dye increases when the membrane potential collapses.^16^ The three best antibacterial AuPt NPs, Au_90_Pt_10_, Au_80_Pt_20_, and Au_66_Pt_34_, can significantly disrupt the inner membrane and decrease the membrane potential (Figure 2 B). We deduced that the collapse of membrane potential probably led to bacterial death, which is in accordance with mechanisms for antibacterial agents.2b, 16, 17

**Figure 2:**
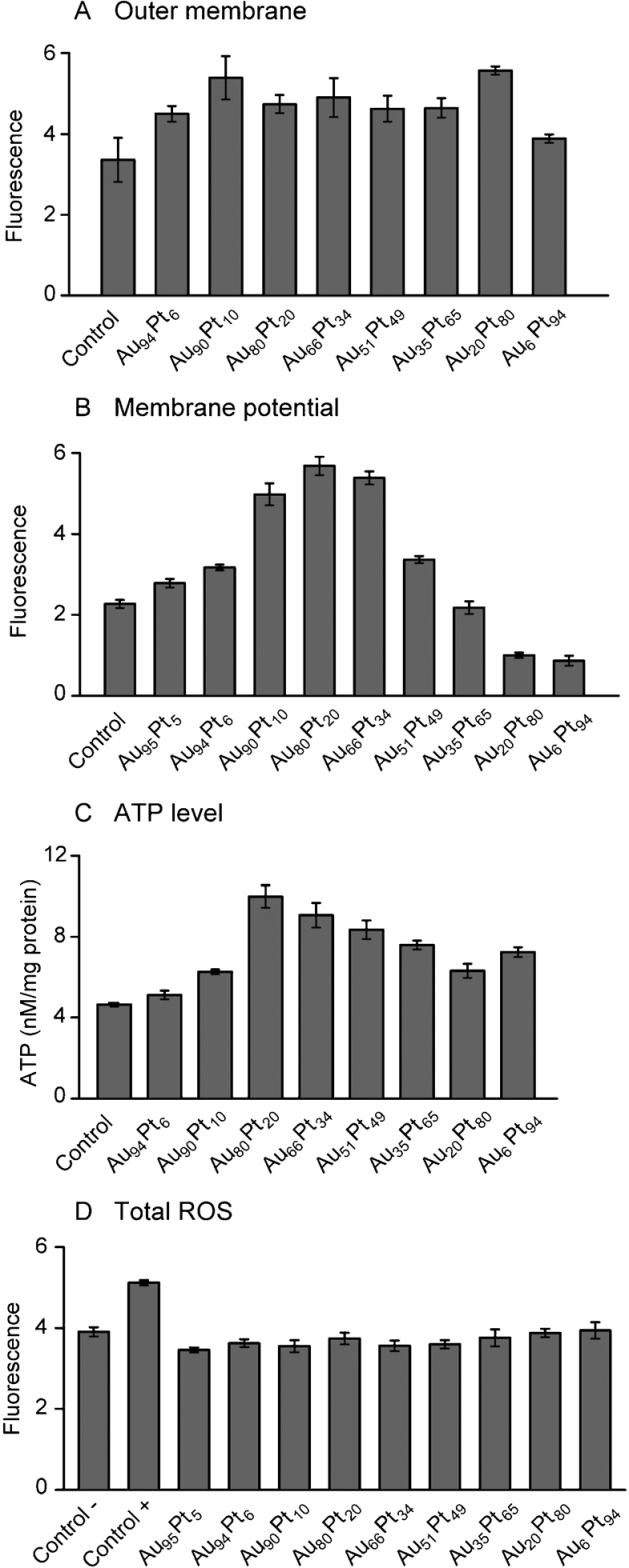
The effects of AuPt NPs on the cell membrane and the respiration chain of *E. coli*. A) Outer membrane permeability probed with the NPN dye. B) Inner membrane potential probed with the DiSC_3_(5) dye. C) Intracellular ATP concentrations corrected with the protein content. D) Cellular total ROS probed with 2′,7′-dichlorofluorescein diacetate (DCFH-DA). *E. coli* without addition of NPs was the control in all assays or the negative control in (D). The positive control in (D) was commercial Rosup producing ROS in the kit.

Because of the vital role ATP plays in bacterial metabolism, we determined the level of intracellular ATP, the activity of F-type ATP synthase, and the NAD^+^/NADH reaction in the inner membrane. The generation of ATP is an important part in the bacterial respiration chain, which requires the membrane potential, F-type ATP synthase, and protons from the NAD^+^/NADH reaction. Surprisingly, we found that AuPt NPs with high antibacterial activities significantly increased intracellular ATP levels, among which Au_80_Pt_20_ induced a 2-fold increase compared to the control (Figure 2 C). By contrast, AuPt NPs reduced the activity of F-type ATP synthase and did not affect the ratio of NAD^+^ to NADH (Figure S3). There are two possibilities that can explain the elevation of ATP levels. One is that AuPt as an alternative enzyme could catalyze the generation of ATP. It has been reported that high ATP levels caused by the overexpression of Pck kinase can inhibit the growth of *E. coli*, and at the same time up-regulate DNA damage-related genes.^18^ High ATP level can thus be toxic to bacteria. The other possibility is that AuPt could inhibit the synthesis of proteins that consume ATP, thus inducing the accumulation of ATP. In this respect, AuPt NPs resemble the antibiotic chloramphenicol, a kind of protein synthesis inhibitor that is shown to increase the level of ATP.^19^ The increase of the level of ATP, in addition to being known in some cases of small-molecular antibiotics, is also a strategy that the human immune system employs to kill bacteria.^20^ ATP-mediated bacterial killing is independent of ROS.^21^

We examined the production of ROS in NP-treated *E. coli* to test if bacterial death resulted from oxidative damage, since AuPt NPs can catalyze some redox reactions. Bactericidal antibiotics can induce the generation of ROS to kill bacteria.^22^ V_2_O_5_ NPs as oxidase mimics have been reported to inhibit the bacterial biofilm via ROS.^23^ Fe_3_O_4_-doped silica NPs show composition- and catalysis-related ROS effects on mammalian cells.^24^ By determining the total ROS with 2′,7′-dichlorofluorescein diacetate (DCFH-DA) and hydroxyl radical (a type of ROS) with hydroxyphenyl fluorescein (HPF), we found that AuPt NPs did not significantly increase the production of either total ROS (Figure 2 D) or hydroxyl radicals (Figure S3). Hence the antibiotic mechanism of bactericidal AuPt NPs does not involve any ROS; this result shows that the mechanisms of action of AuPt NPs are quite different from conventional antibiotics.

We determined the Pt release of AuPt NPs to test if the soluble Pt inside the bacterial cell contributed to bacterial death, since some NPs exert toxicity with their soluble content inside cells.^25^ In neutralophilic bacteria, cytoplasmic pH values are 7.5–7.7.^26^ We incubated 50 μg mL^−1^ of Au_95_Pt_5_, Au_80_Pt_20_, Au_66_Pt_34_, Au_6_Pt_94_ NPs in H_2_O (pH 7.0) and phosphate buffered saline (PBS, pH 7.6) at 37 °C for 96 h, centrifuged with Millipore ultrafilter (MWCO 3000 Da, 1.5 nm or larger NPs cannot pass through the filter), and determined the content of Au and Pt in the filtrate with ICP-MS. The Au release amount is zero for all NPs in H_2_O and PBS (Table S4). The Pt release percentage is less than 0.2 % for all NPs in H_2_O and in PBS. The Pt release amount of NPs is far less than MICs. For example, 0.03 μg mL^−1^ of Pt was released from 50 μg mL^−1^ of Au_80_Pt_20_ while its MIC was 5 μg mL^−1^ against *E. coli*. There is no correlation between the Pt release and the antibacterial activity. For example, the Pt amount released from Au_6_Pt_94_ was nearly identical to that from Au_80_Pt_20_, but Au_6_Pt_94_ was totally inactive. Thus, the Pt release could not be a cause for bacterial death.

Our investigation in the antibacterial mechanism of AuPt NPs shows that their excellent antibiotic activities mainly come from: 1) the damage of the inner membrane that compromises the integrity of bacteria; 2) the increase of the intracellular ATP level. A surprise here is that the antibiotic mechanism does not involve ROS at all. This mechanism is therefore unique, because most commercially available bactericidal antibiotics or metal-based agents (e.g., Ag, Cu, ZnO, TiO_2_) invariably involve elevated levels of ROS to kill bacteria.

For a preliminary evaluation of the potential toxicity of AuPt NPs, we tested their effect on the viability of human umbilical vein endothelial cells (HUVECs) with a CCK-8 kit, which quantifies the number of viable cells. Antibiotic AuPt NPs did not affect the cell viability (>95 %) at the concentration as high as 80 μg mL^−1^ after 24 h of incubation (Figure 3). After the longer-term incubation such as 48 h and 72 h, NPs at 40 μg mL^−1^ did not affect the cell viability (>95 %) (Figure S4). Hence, AuPt NPs showed selective toxicity to bacteria but not to mammalian cells. This selectivity could result from the difficulty for NPs to act on the respiration chain in the mammalian mitochondria, which need to overcome several barriers including the escape from lysosomes, targeting mitochondria, and entry into of mitochondria through the membrane.^27^

**Figure 3:**
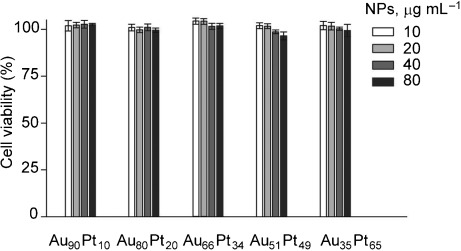
Cell viability of HUVECs after incubation for 24 h with antibiotic AuPt NPs at different concentrations.

In conclusion, our work reports that AuPt NPs are potent antibacterial agents and harmless to human cells. By contrast, pure Au NPs or pure Pt NPs are not antibiotic at all. The antibiotic mechanisms of AuPt NPs include the rupture in the bacterial inner membrane and the increase of intracellular ATP levels, but do not involve the generation of ROS. Further work is required to elucidate the identity of the substrate in the intracellular catalytic reactions related to this process. Nevertheless, we believe that our findings in this work not only extend the application of bimetallic NPs (made of inert noble metals) as new classes of antibacterial agents, but may also provide new perspectives in biological reactions to broaden the application of NPs in medicine.

Dedicated to Professor George M. Whitesides on the occasion of his 75th birthday
