# Supplementary material for: Tuning the Composition of AuPt Bimetallic Nanoparticles for Antibacterial Application
Source: Angew Chem Int Ed Engl. 2014 May 14;53(31):8127–31. doi: 10.1002/anie.201401035 (PMC4320751; doi:10.1002/anie.201401035)
Supplement: Supplementary file 1 [file anie0053-8127-sd1.pdf]

Angewandte Chemie
Eine Zeitschrift der Gesellschaft Deutscher Chemiker

Supporting Information

© Wiley-VCH 2014

69451 Weinheim, Germany

Tuning the Composition of AuPt Bimetallic Nanoparticles for Antibacterial Application**

*Yuyun Zhao, Chunjie Ye, Wenwen Liu, Rong Chen, and Xingyu Jiang**

anie_201401035_sm_miscellaneous_information.pdf

Contents:

Methods including materials, synthesis and characterization of AuPt NPs, antibacterial activity test, preparation of bacterial samples for SEM and TEM, membrane permeability assay, determination of ATP, ATPase, NAD+/NADH, and ROS, Pt release, and cytotoxicity assay.

Figure S1 TEM images of Au, AuPt , and Pt NPs and the statistic diameters of NPs.

Figure S2 UV-Vis spectra of AuPt NPs.

Figure S3 ATPase activity, the ratio of NAD^+/NADH, and cellular HO ·.

Figure S4 Cell viability of HUVECs after incubation for 48 h and 72 h with antibiotic AuPt NPs.

Table S1 Zeta potential of Au, AuPt, and Pt NPs.

Table S2 MICs in broth mixed with 10% fetal bovine serum.

Table S3 Comparison of antibiotic and catalytic activities of AuPt NPs.

Table S4 The Pt release of NPs in H_2O (pH 7.0) and PBS (pH 7.6)

Materials. $HAuCl_4$ $3H_2O$, K_2PtCl_4, and $NaBH_4$ were from Sinopharm Chemical Reagent Beijing Co., Ltd. Tween 80 were from MP Biomedical. NPN and DiSC3(5) were from Molecular Probes. Mueller Hinton broth was from Oxoid. The ATP assay kit, total ROS assay kit, hydroxyl radical assay kit, and CCK-8 kit were from Beyotime Institute of Biotechnology, China. The ATPase assay kit was from Genmed Scientifics Inc., China). *Escherichia coli* ATCC 11775, *Pseudomonas aeruginosa* ATCC 27853, and *Salmonella choleraesius* ATCC14028 were from China General Microbiological Culture Collection Center. *Klebsiella pneumoniae* ATCC13883 was from Agricultural Culture Collection of China. Clinical isolate MDR *E. coli* BJ915 was from a domestic hospital in China. The human umbilical vein endothelial cell line (HUVEC) was a gift from Prof. Xiyun Yan at Institute of Biophysics, Chinese Academy of Sciences. Dulbecco's modified Eagle medium (DMEM) with high glucose without phenol red and fetal bovine serum (FBS) were from Gibco.

Synthesis and characterization of NPs. We stirred the mixture of $HAuCl_4$ $3H_2O$ (0.01 mmol), varied amounts of K_2PtCl_4, and 40 mg Tween 80 in 10 mL water for 30 min in the ice-water bath, added $NaBH_4$ ([3 mol $NaBH_4$ / 1 mol $HAuCl_4$ + 2 mol $NaBH_4$/ 1 mol K_2PtCl_4] × 1.2 mol) dropwise with vigorous stirring. After 10 min, we stirred the solution gently for an hour in the ice-water bath. We dialyzed the solution (14 kDa molecular weight cut off) for 24 h against the deionized water, and stored the aqueous solution of AuPt NPs at 4 ^oC for use. Au NPs and Pt NPs were synthesized using a similar method. The compositions of AuPt NPs were determined with the inductively coupled plasma - optical emission spectroscopy (ICP-OES). We observed the morphology of NPs with TEM (Tecnai G^2 20). We determined UV-Vis absorption of the NPs solution with UV-2450 (shimadzu) spectrophotometer and zeta potential values with Zetasizer Nano ZS (Malvern Instruments).

Antibacterial activity test. Bacteria were cultured in the LB medium at 37^oC on a shaker bed at 200 rpm until the logarithmic phase. We added 100 μL of NPs at different concentration in the MH broth in a 96-well microplate, added 10 μL of bacterial suspension (the final density of bacteria was 2-5 $\times 10^4$ CFU/mL) in each well, and incubated them at 37 ^oC for 24 h. The MIC was the concentration at which no visible bacteria grew. MBC was tested by the further culture of the bacterial suspension after testing MIC on LB agar plates at 37 ^oC overnight. MBC was the lowest concentration of NPs where the bacterial colony was counted lower than five. MIC test in broth mixed with 10% FBS was carried out according to the above procedure. Bacteria grew too poorly to obtain MICs in the DMEM (with high glucose without phenol red) with 10% FBS.

Preparation of bacterial samples for SEM and TEM. *E. coli* in the logarithmic phase was mixed with NPs at the final concentration of 40 μg/mL and cultured for 2 h at 37 ^oC on a shaker bed at 200 rpm. We collected bacteria with centrifugation at 8000 rpm for 3 min and fixed them with 2.5% glutaraldehyde overnight at 4 ^oC. We washed them with PBS for three times, dehydrated them through graded ethanol solutions. Finally, we put the samples on the silicon glide and observed SEM. The superthin slices for TEM were prepared according to the reported method. [1]

Membrane permeability assay. For outer membrane assay, we collected samples after the treatment of *E. coli* with 40 μg/mL NPs similar to the above step, dispersed them in PBS (0.01 M, pH 7.4), incubated them with an equal volume of the NPN solution (final concentration is 10 μM) in the dark for 30 min at the room temperature, washed them with PBS twice, and tested the fluorescence intensity under the excitation at 350 nm and the emission at 420 nm. For inner membrane potential, we collected samples after the treatment of *E. coli* with 0.6 μg/mL NPs similar to the above step, diluted them with the HEPES/glucose buffer (5 mM HEPES, 5 mM glucose, pH 7.0) to an OD_{600nm} of 0.05, incubated them with 4 μM DiSC3-5 for 1 h, immediately ion-equilibrated with 100 mM KCl for 1 h, and the fluorescence was monitored with excitation at 622 nm and emission at 670 nm.

ATP level. We treated mid-logarithmic phase *E. coli* with 10 μg/mL of AuPt NPs at 37 ^oC for 4 h and collected bacteria. The treatment procedure was also applied to the following ATPase assay. We extracted ATP and tested the content using the commercial ATP assay kit (Beyotime Institute of Biotechnology, China). We added the lysis buffer (contained in the kit) in bacterial cells and centrifuged them at 12000 rpm at 4 ^oC for 5 min. The supernatant was separated to two parts. To one part we added the ATP-detecting solution (contained in the kit) and immediately determined the luminescence with the Tecan infinite 200 microplate reader. To the other part we quantified the protein content with the BCA protein assay kit (Beyotime Institute of Biotechnology, China) and corrected the ATP level for different samples.

ATPase activity. We extracted membrane proteins and determined the activitie of F-type ATP synthase using the F-type ATPase activity assay kit. We added the lysis buffer (Reagent A in the kit) in bacterial cells in the ice bath, incubated them for 30 min, votexed them for 6 min three times, centrifuged them at 1000 rpm at 4 ^oC for 10 min. The clear supernatant was transferred in the new tubes and centrifuged at 16000 rpm at 4 ^oC for 15 min. We removed the supernatant and added Reagent B in the precipitate (we called "the sample") for the ATPase activity assay and the protein amount assay. We incubated Reagent C, D, and F at 30 ^oC for 3 min and mixed it with the sample quickly. The difference in value between the absorbance at the zero time and the absorbance at 5 min was used to calculate the total activity (A_{total}) according to the formula provided by the kit. We used Reagent E instead of the sample as the negtive control ($A_{negtive}$). We added Reagent G in the sample and tested the activity as the non-specific activity (A_{ns}). The sample activity was the value of (A_{total} - A_{ns}).

NAD^+/NADH. We treated mid-logarithmic phase *E. coli* with 10 μg/mL of AuPt NPs at 37 ^oC for 3 h, during which time we took 1 mL of samples every half hour of culturing to extract and determine NAD^+ and NADH according to the reported procedure. [2] We centrifuged the samples at 13000 rpm for 1 min, removed the supernatant, and froze the precipitate immediately in a -80 ^oC refrigerator until all time-point samples were collected. We added 75 μL of 0.2 M NaOH (for the NADH extraction) or 75 μL of 0.2 M HCl (for the NAD^+ extraction) to the ice-cold precipitate, heated it at 100 ^oC for 10 min, and centrifuged it at 10000 rpm for 5 min. The supernatant containing NAD^+/NADH was stored on ice in the dark. The reaction mixture of NAD cycling assay was composed of 16 μL of 1.0 M bicine (pH 8.0), 40 μL sample extract, 40 μL neutralizing buffer (0.1 M HCl for NADH, or 0.1 M NaOH for NAD^+), 16 μL of 16.6 mM phenazine ethosulfate (PES), 16 μL of 4.2 mM MTT (3-[4, 5-dimethylthiazol-2-yl]-2, 5- diphenyltetrazolium bromide), 16 μL of pure alcohol, and 30 μL of 40 mM EDTA (pH 8.0). We added PES and MTT to the samples containing NAD^+/NADH in the 96-well plate, incubated them for 3 min at 30 ^oC, and added 3.2 μL of alcohol dehydrogenase (500 U/mL, in the bicine buffer, pH 8.0). We monitored the change in absorbance at 570 nm during 10 min at 30 ^oC. The reduction rate of MTT is proportional to the concentration of NAD^+ or NADH. NAD^+ and NADH standard products (Sigma) with concentrations of 0.0375 to 0.75 nM were used to calibrate the assay.

ROS assay. For total ROS, we stained samples with 10 μM 2',7'-dichlorodihydrofluorescein diacetate (DCFH-DA) for 30 min in the dark at the room temperature and washed them with PBS once according to total ROS assay kit. Rosup producing ROS in the kit was the positive control. We measured the fluorescence with the Tecan infinite 200 microplate reader with excitation at 488 nm and emission at 525 nm.

For hydroxyl radicals, we stained samples with 5 μM hydroxyphenyl fluorescein (HPF) for 30 min in the dark at the room temperature and washed them with PBS once according to the hydroxyl radical kit. The fluorescence was analyzed by a flow cytometer (BD FACSCalobur) with excitation at 488 nm and emission at 515 nm, where 30,000 cells were measured for each sample. The data was analyzed using FlowJo.

Pt release. We incubated 50 μg/mL of $Au_{95}Pt_5$, $Au_{80}Pt_{20}$, $Au_{66}Pt_{34}$, Au_6Pt_{94} NPs in H_2O (pH 7.0) and phosphate buffered saline (PBS, pH 7.6) at 37 ^oC for 96 h, centrifuged first with Millipore ultrafilter (MWCO 10000 Da, 3 nm or larger NPs cannot pass through the filter) then with Millipore ultrafilter (MWCO 3000 Da, 1.5 nm or larger NPs cannot pass through the filter), and determined Au and Pt content in the filtrate with ICP-MS (Perkinelmer NexION 300X).

Cytotoxicity assay. HUVEC cells were cultured in the Dulbecco's modified Eagle medium (DMEM) containing 10% fetal bovine serum. We incubated 1×10^4 cells per well of HUVEC in 96-well plates with different concentrations of AuPt NPs in 200 μL of medium at 37 ^oC for 24 h, 48 h, and 72 h, washed the cells once with PBS (0.01 M, pH 7.4), added 10% (v/v) of the CCK-8 solution in the medium, incubated the sample at 37 ℃ for 2 h, and determined the absorbance at 450 nm referred with 650 nm with the Tecan infinite 200 microplate reader.

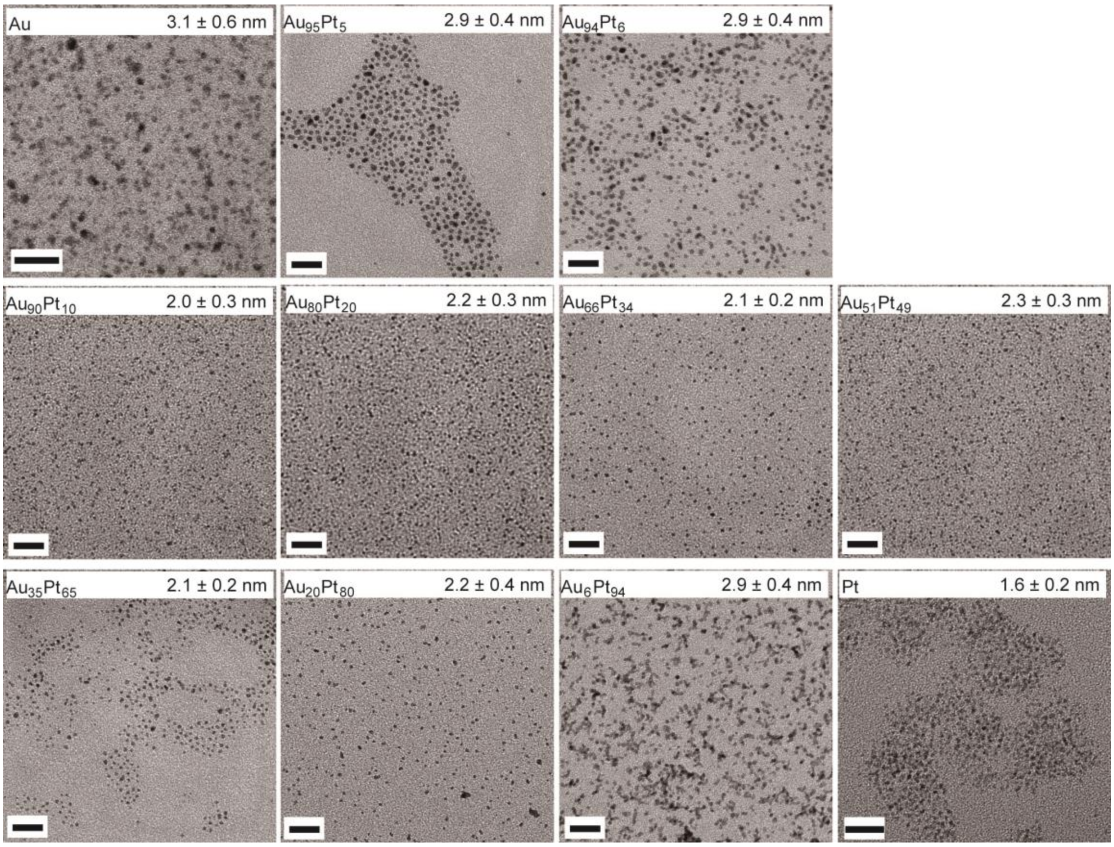

Figure S1. TEM images and statistic diameters of Au, AuPt, and Pt NPs. Scale bars: 20 nm.

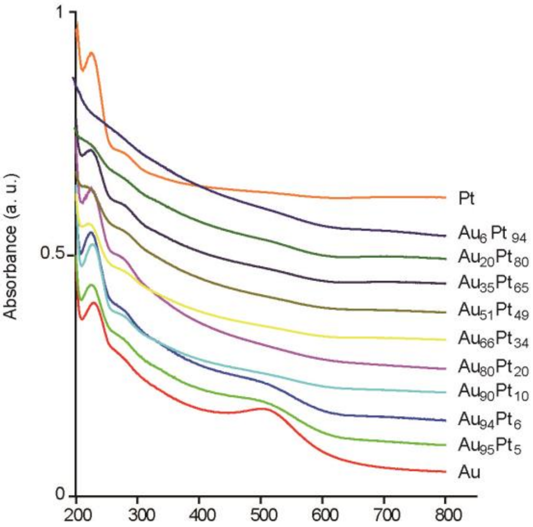

Figure S2. UV-Vis spectra of AuPt, Au, and Pt NPs. The characteristic absorption of Au disappeared with the increase of Pt contents in AuPt NPs. To clearly show the absorption of each NP, we offset the lines vertically.

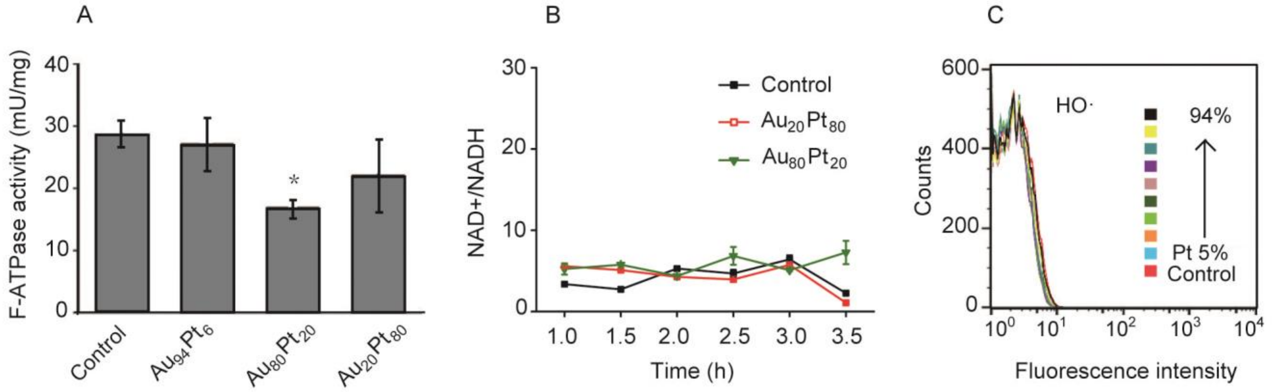

Figure S3. ATPase activity (A), the ratio of NAD^+/NADH (B), and cellular HO· (C). *E. coli* without treatment of NPs was the control. Antibacterial $Au_{80}Pt_{20}$ significantly decreased the activity of F-ATPase ($P < 0.01$). AuPt NPs did not affect the reaction of charge transfer chain (the ratio of NAD^+ to NADH), nor induced the production of hydroxyl radicals in bacterial cells. It is different from the reported bactericidal antibiotics. [3]

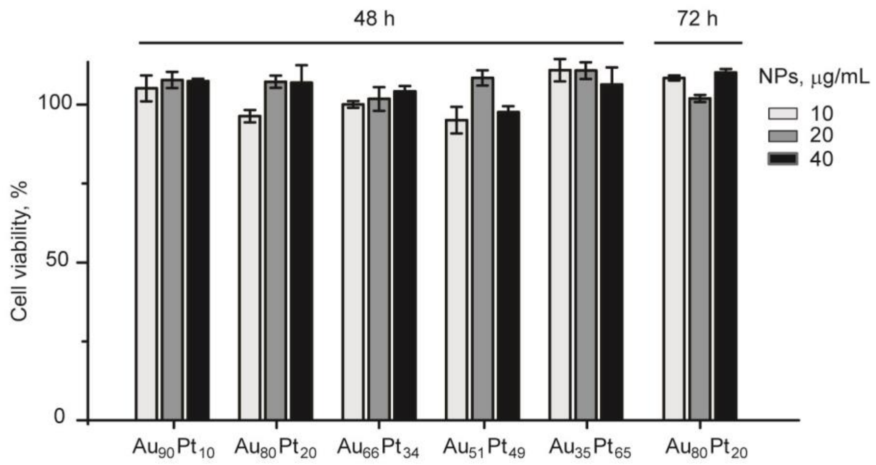

Figure S4. Cell viability of HUVECs after incubation for 48 h and 72 h with antibiotic AuPt NPs at different concentrations.

Table S1. Zeta potential of Au, AuPt, and Pt NPs in water at 25 ^{o}C.

NPs	Zeta potential (mV)
Au	-15.4 ± 2.7
$Au_{95}Pt_5$	-16.0 ± 1.2
$Au_{94}Pt_6$	-14.1 ± 2.5
$Au_{90}Pt_{10}$	-9.6 ± 1.5
$Au_{80}Pt_{20}$	-12.7 ± 1.8
$Au_{66}Pt_{34}$	-12.7 ± 1.5
$Au_{51}Pt_{49}$	-27.1 ± 2.4
$Au_{35}Pt_{65}$	-29.2 ± 2.5
$Au_{20}Pt_{80}$	-36.9 ± 1.6
Au_6Pt_{94}	-40.4 ± 0.9
Pt	-17.3 ± 1.1

Table S2. MICs in broth mixed with 10% fetal bovine serum (FBS). Bacteria grew too poorly to obtain MICs in the DMEM with 10% FBS.

		MIC, μg/mL		
		E. coli	MDR *E. coli*	*K. p*
Standard broth	$Au_{80}Pt_{20}$	5	5	5
	$Au_{66}Pt_{34}$	6	6	12
Broth + 10% FBS	$Au_{80}Pt_{20}$	16	4	8
	$Au_{66}Pt_{34}$	16	8	8

Table S3. The antibiotic and catalytic activities of AuPt NPs of different compositions.

$Au_{100-x}Pt_x$ [a]	Antibiotic, MIC [b]	$Au_{100-x}Pt_x$	Catalytic, ip [c]
Au	>128	-	-
$Au_{95}Pt_5$	>105	-	-
$Au_{94}Pt_6$	>39	$Au_{93}Pt_7$	0.21
$Au_{90}Pt_{10}$	9	$Au_{85}Pt_{15}$	0.26
$Au_{80}Pt_{20}$	6	$Au_{77}Pt_{23}$	0.57
$Au_{66}Pt_{34}$	8	$Au_{68}Pt_{32}$	0.56
$Au_{51}Pt_{49}$	19	$Au_{60}Pt_{40}$	0.46
$Au_{35}Pt_{65}$	37	$Au_{38}Pt_{62}$	0.37
$Au_{20}Pt_{80}$	> 83	$Au_{27}Pt_{73}$	0.26
Au_6Pt_{94}	>128	$Au_{14}Pt_{86}$	0.21
Pt	>128	-	-

[a] x is the atomic percentage of Pt in the NP. [b] Antibiotic activity is indicated with the MIC (μg/mL) averaged from MICs against five bacteria in Table 1. [c] Peak current values (A) of methanol oxidation reaction per mg of Pt in the NP, which are estimated in the Figure S6 from the literature reported by D. Jeyakumar and co-workers. [4] The lower MIC shows the higher antibiotic and the higher ip shows the higher catalytic activity. The most potent antibiotic AuPt NPs are also the best catalysts, which is highlighted in red in the table.

Table S4. The release amount and percentage of Au and Pt from 50 μg/mL of AuPt NPs in H_2O (pH 7.0) and PBS (pH 7.6) at 37 ^{o}C for 96 h. The Pt release amount of NPs is far less than MICs. And there is no correlation between the Pt release and the antibacterial activity. For example, the Pt amount released from Au_6Pt_{94} was nearly identical to that from $Au_{80}Pt_{20}$, but Au_6Pt_{94} was totally inactive. Thus, the Pt release could not be a cause for the bacterial death.

	In H_2O				In PBS			
	Au, ng/mL	Pt, ng/mL	Au, %	Pt, %	Au, ng/mL	Pt, ng/mL	Au, %	Pt, %
$Au_{95}Pt_5$	0	0.2 ± 0.0	0	0	0	5.1 ± 0.0	0	0.01
$Au_{80}Pt_{20}$	0	3.3 ± 0.0	0	0.01	0	35.5 ± 0.2	0	0.07
$Au_{66}Pt_{34}$	0	8.8 ± 0.1	0	0.02	0	91.1 ± 1.5	0	0.18
Au_6Pt_{94}	0	37.7 ± 0.3	0	0.08	0	35.8 ± 0.2	0	0.07

References

[1] Y. Zhao, Y. Tian, Y. Cui, W. Liu, W. Ma, X. Jiang, *J. Am. Chem. Soc.* **2010**, *132*, 12349-12356.
[2] Y. Cui, Y. Zhao, Y. Tian, W. Zhang, X. Lu, X. Jiang, *Biomaterials* **2012**, *33*, 2327-2333.
[3] M. A. Kohanski, D. J. Dwyer, B. Hayete, C. A. Lawrence, J. J. Collins, *Cell* **2007**, *130*, 797-810.
[4] N. Ilayaraja, N. Prabu, N. Lakshminarasimhan, P. Murugan, D. Jeyakumar, *J. Mater. Chem. A* **2013**, *1*, 4048-4056.
